# Modification of single-nucleotide polymorphism in a fully humanized CYP3A mouse by genome editing technology

**DOI:** 10.1038/s41598-017-15033-0

**Published:** 2017-11-09

**Authors:** Satoshi Abe, Kaoru Kobayashi, Asami Oji, Tetsushi Sakuma, Kanako Kazuki, Shoko Takehara, Kazuomi Nakamura, Azusa Okada, Yasuko Tsukazaki, Naoto Senda, Kazuhisa Honma, Takashi Yamamoto, Masahito Ikawa, Kan Chiba, Mitsuo Oshimura, Yasuhiro Kazuki

**Affiliations:** 1 Chromosome Engineering Research Center (CERC), Tottori University, 86 Nishi-cho, Yonago, Tottori, 683-8503 Japan; 2 Graduate School of Pharmaceutical Sciences, Chiba University, 1-8-1 Inohana, Chuo-ku, Chiba, 260-8675 Japan; 3 Research Institute for Microbial Diseases, Osaka University, Suita, Osaka, 565-0871 Japan; 4 Department of Mathematical and Life Sciences, Graduate School of Science, Hiroshima University, Higashi, Hiroshima, 739-8526 Japan; 5 Division of Laboratory Animal Science, Research Center for Bioscience and Technology, Tottori University, 86 Nishi-cho, Yonago, Tottori, 683-8503 Japan; 6 Tsukuba Bioanalytical Laboratory, Shin Nippon Biomedical Laboratories, Ltd., 2-1-6 Sengen, Tsukuba, Ibaraki, 305-0047 Japan; 7 Department of Biomedical Science, Institute of Regenerative Medicine and Biofunction, Graduate School of Medical Sciences, Tottori University, 86 Nishi-cho, Yonago, Tottori, 683-8503 Japan

## Abstract

Cytochrome P450, family 3, subfamily A (CYP3A) enzymes metabolize approximately 50% of commercially available drugs. Recently, we developed fully humanized transchromosomic (Tc) CYP3A mice with the CYP3A cluster including *CYP3A4*, *CYP3A5*, *CYP3A7*, and *CYP3A43*. Our humanized CYP3A mice have the CYP3A5*3 (g.6986G) allele, resulting in the almost absence of CYP3A5 protein expression in the liver and intestine. To produce model mice for predicting CYP3A5′s contribution to pharmacokinetics, we performed a single-nucleotide polymorphism (SNP) modification of *CYP3A5* (g.6986G to A, *3 to *1) on the CYP3A cluster using genome editing in  both mouse ES cells and fertilized eggs, and produced humanized CYP3A5*1 mice recapitulating the CYP3A5*1 carrier phenotype in humans. The humanized CYP3A mouse with CYP3A5*1 is the first Tc mouse for predicting the SNP effect on pharmacokinetics in humans. The combination of Tc technology and genome editing enables the production of useful humanized models that reflect humans with different SNPs.

## Introduction

Cytochrome P450, family 3, subfamily A (CYP3A) is a group of drug-metabolizing enzymes that metabolize approximately 50% of commercially available drugs. Therefore, in the field of drug development, the contribution of CYP3A in pharmacokinetics of new chemical entities has to be evaluated. In conventionally used experimental animals, such as mice and rats, the conditions do not completely reflect the human pharmacokinetics of drugs metabolized by CYP3A. It is well known that substantial species differences exist in CYP3A enzymes^1^. Therefore, we developed a humanized transchromosomic (Tc) CYP3A mouse models based on human artificial chromosome (HAC) and mouse artificial chromosome (MAC) vectors using chromosome engineering technology.

CYP3A genes are clustered together on human chromosome 7 and include *CYP3A4*, *CYP3A5*, *CYP3A7*, and *CYP3A43*. We cloned 700 kilobases, including the *CYP3A* cluster on human chromosome 7, into the HAC vector (CYP3A-HAC) and transferred the CYP3A-HAC into mouse embryonic stem (ES) cells via microcell-mediated chromosome transfer (MMCT). We produced humanized mice carrying the CYP3A-HAC with a mouse-Cyp3a-knockout (KO) background. Our models recapitulated the gender-, tissue-, and developmental-stage-specific CYP3A expression and the CYP3A-related drug metabolism in humans^2–4^. Recently, we generated novel fully humanized CYP3A (CYP3A-MAC) mice with almost same characteristics with the CYP3A-HAC mice excepting the stability of the exogenous chromosome by using a MAC vector which is more stably maintained in mice than the HAC^5,6^ (Kazuki Y. *et al.*, unpublished data).

Studies on the contributions of CYP3A enzymes to pharmacokinetics have mainly focused on CYP3A4 in adults. CYP3A7 expressed in fetuses is gradually replaced by CYP3A4 after birth^7,8^. Although little is known about the function of CYP3A43, the importance of the contributions of other CYP3As to pharmacokinetics, particularly CYP3A5, should also be considered. Despite CYP3A5 being highly similar to CYP3A4 at the amino acid level, the degrees of catalytic capability and regioselectivity toward some substrates are different^9–11^. *CYP3A5* genotypes are associated with the dosing condition of many drugs, such as tacrolimus, which is a well-known immune suppressor used for organ transplantation^12,13^. Moreover, it has been reported that CYP3A5 contributes to therapy resistance in different subtypes of pancreatic ductal carcinoma^14^. Therefore, the contribution of CYP3A5 to drug metabolism and the mechanism of expression of CYP3A5 have been re-evaluated recently.

*CYP3A5* single-nucleotide polymorphisms (SNPs) have been known to affect CYP3A5 expression. Among various SNP alleles of the CYP3A5 gene that have been reported, the most well-studied ones are CYP3A5*1 (6986A) and CYP3A5*3 (g.6986A > G)^15–17^. SNP associated with these alleles is located in intron 3 of *CYP3A5*. Carriers of the CYP3A5*1 allele express the CYP3A5 protein, while homozygotes of the CYP3A5*3 allele show a splicing defect resulting in the absence of CYP3A5 protein expression. It has been reported that 10–30% of adult Caucasians and Asians express detectable amounts of CYP3A5, whereas 60% of African-Americans do so. Taking these findings together, the prediction of CYP3A5-related drug pharmacokinetics in both CYP3A5 expressers and non-expressers is very important. Humanized model animals are thought to be useful in pursuit of this goal. However, the CYP3A5 genotype of our humanized CYP3A model mice was CYP3A5*3, which was associated with undetectable protein expression in the liver and very low protein expression in the intestine. Therefore, the contribution of CYP3A5 could not be evaluated in this model. Although there are compounds metabolized by both CYP3A4 and CYP3A5, the extent to which CYP3A5 contributes to the metabolism of such substrates *in vivo* remains unclear. Thus, a model that expresses both CYP3As, CYP3A4 and CYP3A5, is expected to be useful in the validation of contribution. To confirm the contribution of human CYP3A5 to drug metabolism using a model mouse, the development of a model harboring the human CYP3A5*1(6986A) allele was desired.

Recently, genome editing technologies, such as zinc-finger nuclease, transcription activator-like effector nuclease, and clustered regularly interspaced short palindromic repeats/Crispr associated protein 9 (CRISPR/Cas9), have provided more efficient options to modify a targeted sequence for the production of KO or knock-in (KI) cells and animals^18^. These technologies generate DNA double-strand breaks (DSBs). DSBs are repaired by either non-homologous end joining or homology-directed repair (HDR) machinery. An error in the repair process disrupts the targeted gene or sequence, resulting in the production of KO cells and organisms. If DSBs are induced in the presence of double-stranded or single-stranded donor DNA with a homologous sequence, we can obtain KI cells and animals or achieve the desired modification of the sequence. Currently, the CRISPR/Cas9 system is the most convenient tool for making desired modifications to targeted sequences.

In the present study, we produced humanized CYP3A5*1 mice by modifying the CYP3A5*3 SNP on the CYP3A-MAC using genome editing technology, namely, the CRISPR/Cas9 system. We successfully modified CYP3A5*3 SNP to CYP3A5*1 (6986G to A) in both mouse ES cells (ESC-transfection) and fertilized eggs (pronuclear injection), for which the modification efficiencies were high. Expression of the CYP3A5 protein in the liver and intestine was higher in humanized CYP3A5*1 mice than in CYP3A5*3 mice. The contribution of CYP3A5 was also evaluated by inhibition assays, and higher CYP3A5 enzymatic activity was detected in the liver and intestinal microsomes of CYP3A5*1 mice than in those of CYP3A5*3 mice. The humanized CYP3A (CYP3A5*1) mice are useful for evaluating the contribution of human CYP3A5 to drug screening and for understanding the mechanism of *CYP3A5* gene expression.

## Results

### Validation of CRISPR/Cas9 gRNA for targeted cleavage

Previously, we generated CYP3A-HAC mouse using the HAC vector. Recently, we successfully generated CYP3A-MAC and Tc mice with CYP3A-MAC via mouse ES cells (the manuscript is in preparation). Because the MAC is more stably maintained in mice than the HAC, we utilized the CYP3A-MAC in mouse ES cells and fertilized eggs in the present study.

**Figure 1:**
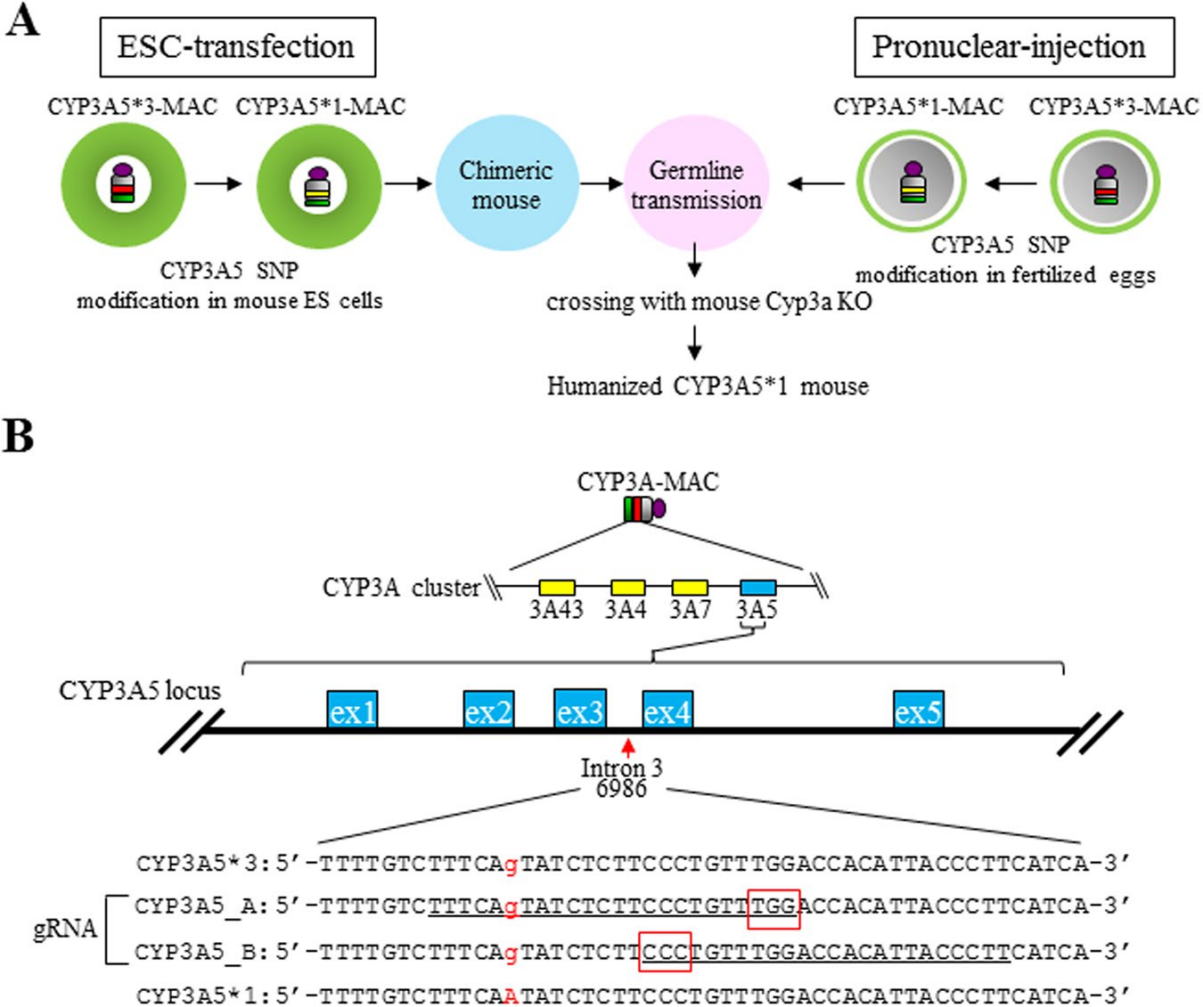
Strategy of humanized CYP3A5*1 mouse production and the design of the target for SNP modification. (**A**) Schematic diagram of humanized CYP3A5*1 mouse development. SNP of *CYP3A5* on the CYP3A-MAC was modified in both mouse ES cells and fertilized eggs. Mice with the CYP3A*1-MAC were mated with mouse-Cyp3a-KO mice, and humanized CYP3A5*1 mice were produced. (**B**) The map of *CYP3A5* SNP locus. CYP3A5*1 and CYP3A5*3 sequence and CRISPR/Cas9 targeting sites, CYP3A5_A and CYP3A5_B, are underlined. Red characters show SNPs and red rectangles indicate PAM sequences.

The *CYP3A5* genotype on the CYP3A cluster was CYP3A5*3, as described previously^2^. To modify *CYP3A5* SNP on the CYP3A-MAC, we adapted an HDR-mediated modification strategy using the CRISPR/Cas9 system in both mouse ES cells and fertilized eggs (Fig. 1A). In this strategy, cleavage of the target site by the CRISPR/Cas9 system will activate the DNA repair machinery, and HDR-mediated SNP modification will occur in the presence of the donor plasmid or single-stranded oligodeoxynucleotide (ssODN), with a homology sequence harboring the desired SNP. We utilized the pX330 plasmid expressing the Cas9 protein and gRNA for genome editing^19^.

Two target sequences were chosen and two pX330 plasmids were constructed (Fig. 1B). The cleavage activity was first validated using a luciferase gene reconstruction-based single-strand annealing (SSA) assay^20^. The gRNA targeting CYP3A5_B (pX330-B) showed higher activity than that targeting CYP3A5_A (pX330-A) (data not shown). Next, specific on-target cleavage was evaluated using the pCAG-EGxxFP-based SSA assay because a high homology potential off-target sequence exists in other CYP3A genes (*CYP3A4*, *CYP3A7*, and *CYP3A43*) on the CYP3A-MAC (Supplementary Fig. 1)^21^.

The pCAG-EGxxFP plasmid comprises 5′EGFP and 3′EGFP sequences with some overlap between them. The target sequence is inserted between 5′EGFP and 3′EGFP. When the pCAG-EGxxFP-target and pX330-gRNA are co-transfected into culture cells and pX330-sgRNA has the ability to cleave the target sequence, the cleaved EGxxFP-target is repaired by either homologous recombination or SSA, resulting in EGFP reconstitution. Therefore, the cleavage activity can be validated by detecting the number of EGFP-positive cells and the intensity of the EGFP fluorescence signal.

**Figure 2:**
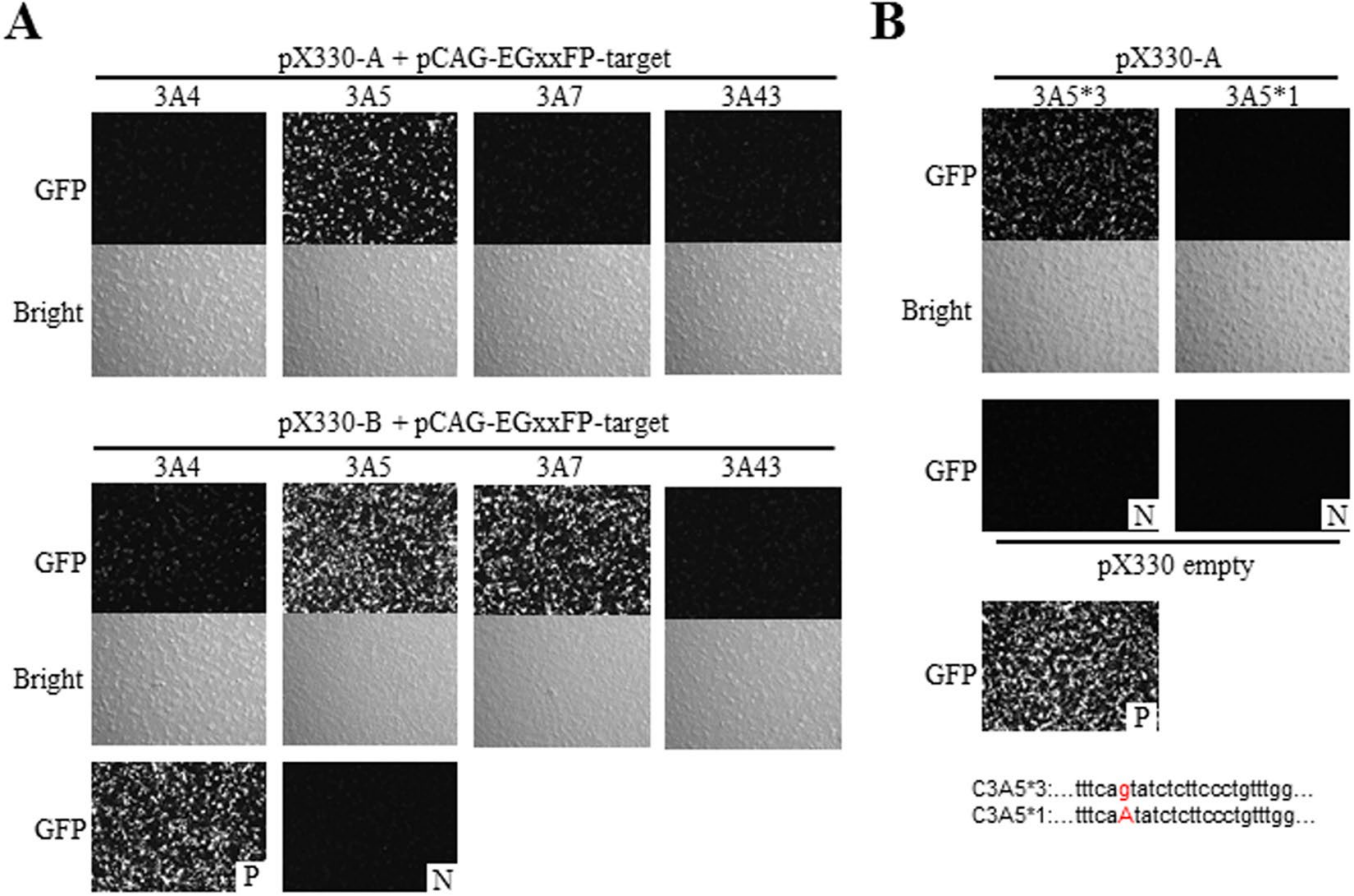
Validation of cleavage activity by the pCAG-EGxxFP system. (**A**) pCAG-EGxxFP assay results for pX330-A and pX330-B activity in the *CYP3A5* region and other off-target sites of each CYP3A region. GFP intensity and GFP-positive cells display the activity. (**B**) pCAG-EGxxFP assay results of pX330-A for validation of the cleavage activity toward the modified sequence. P: positive control with pX330-gRNA and pCAG-EGxxFP-target. N: negative control with pX330 empty and pCAG-EGxxFP-target.

The pCAG-EGxxFP assay revealed that pX330-B has off-target effects on other CYP3A sequences, particularly *CYP3A7* and *CYP3A4*, whereas pX330-A displays specific on-target activity only to *CYP3A5*, without any off-target effects on other CYP3A genes (Fig. 2A). Further analysis was performed on whether pX330-A has the ability to cleave the modified sequence. If it does, the efficiency of the desired modification would be expected to be lower. However, pX330-A did not show any activity toward the expected modified sequence with a 1-bp mismatch (Fig. 2B). Considering these results, we chose to use pX330-A for SNP modification.

### SNP modification in mouse ES cells carrying the CYP3A-MAC and CYP3A5*1 mouse production

**Figure 3:**
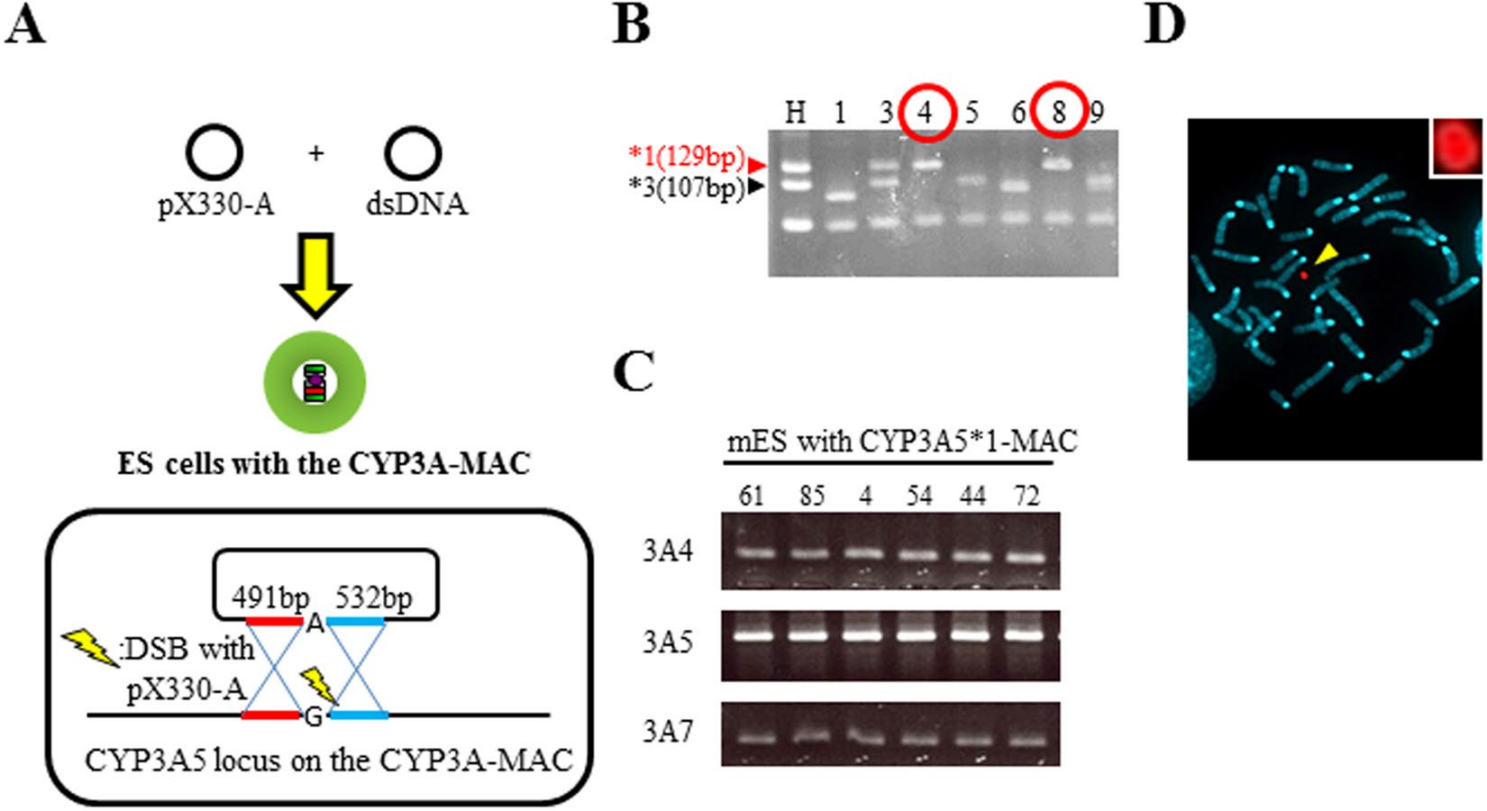
*CYP3A5* SNP modification in mouse ES cells carrying the CYP3A-MAC. (**A**) Schematic diagram of *CYP3A5* SNP modification in mouse ES cells. The pX330-A plasmid and a plasmid with CYP3A5 *1 SNP and each homologous arm of about 500 bp were co-transfected into mouse ES cells carrying the CYP3A-MAC. (**B**) Representative PCR-RFLP assay result. Each sample was numbered. The upper 129-bp and lower 107-bp bands indicate *1 allele and *3 allele, respectively. Red circles indicate candidates. H: human DNA sample with both *1 and *3 alleles as a control. (**C**) Genomic PCR results for the CYP3A region in sequence-positive (*1 allele) clones. (**D**) FISH analysis result of mouse ES cells carrying the CYP3A5*1-MAC. The arrowhead shows the CYP3A5*1-MAC, indicated in red. The inset displays an enlarged image of the CYP3A5*1-MAC.

To produce humanized CYP3A5*1 mice, we performed SNP modification of the CYP3A-MAC in mouse ES cells as described previously^22^. As a donor for the SNP modification of *CYP3A5* in mouse ES cells containing CYP3A-MAC, we employed a plasmid comprising a 500-bp homology arm L, SNP, and a 500-bp homology arm R (HA plasmid) (Fig. 3A). We co-transfected pX330-A with HA plasmids and analyzed 64 clones by PCR-restriction fragment length polymorphism (PCR-RFLP) for *CYP3A5* SNP (Fig. 3B and Supplementary Fig. 2). In total, 12 of 64 clones (18.8%) showed only the CYP3A*1 genotype. The others mainly showed a mixture of cells having the CYP3A5*1 or CYP3A5*3 genotype and a clone without modification (CYP3A5*3). By direct sequencing, we confirmed six clones (9%) with the exact modification of the CYP3A5*1 sequence on the CYP3A-MAC (designated as CYP3A5*1-MAC). Further genomic PCR analyses revealed that other CYP3A genes on the CYP3A-MAC were not disrupted (Fig. 3C and Supplementary Fig. 4). Karyotype analysis confirmed that all six clones had a normal karyotype with independent CYP3A5*1-MAC at a high rate (Fig. 3D). Following this, we produced chimeric mice by injecting mouse ES cells carrying the CYP3A5*1-MAC. The chimeric mice were crossed with mouse-Cyp3a-KO mice, and germline transmission of the CYP3A5*1-MAC was confirmed. Further crossing with mouse-Cyp3a-KO mice eventually produced humanized CYP3A5*1 mice with a Cyp3a-KO background (designated as CYP3A5*1 mouse).

### SNP modification in fertilized eggs carrying the CYP3A-MAC and CYP3A5*1 mouse production

**Figure 4:**
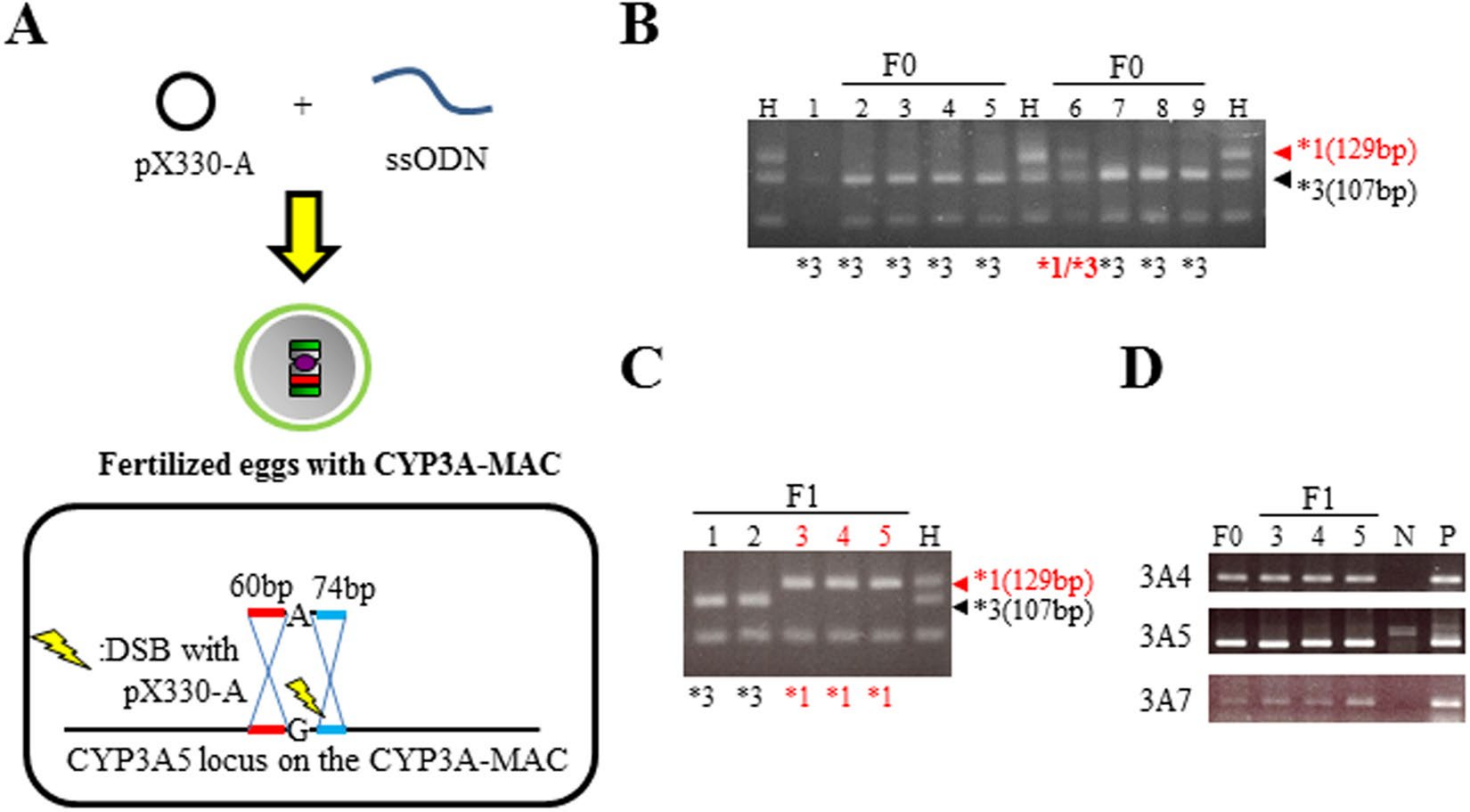
*CYP3A5* SNP modification in fertilized eggs with the CYP3A-MAC by pronuclear injection and the germline transmission of the CYP3A5*1-MAC. (**A**) Schematic diagram of *CYP3A5* SNP modification in fertilized eggs. pX330-A and ssODN with *1 SNP and short homologous arms were injected into fertilized eggs with the CYP3A-MAC. (**B**) PCR-RFLP assay result of F_0_ mice for *CYP3A5* SNP genotyping. (**C**) PCR-RFLP assay result of F_1_ mice. (**D**) Genomic PCR analysis results of the parent mosaic F_0_ mouse and F_1_ mice carrying the CYP3A5*1-MAC. N: WT mouse DNA. P: DNA derived from mouse with the CYP3A5*3-MAC. H: human DNA sample with both *1 and *3 alleles as a control.

For modification of *CYP3A5* SNP, we performed genome editing of the CYP3A-MAC in fertilized eggs. ICR female and male mice carrying the CYP3A-MAC were mated, and then the fertilized eggs were obtained. pX330-A and ssODNs (135 bp containing A) were co-injected into the fertilized eggs (Fig. 4A). Nine mice with GFP positivity (9 of 27; 33%), indicating the presence of the CYP3A-MAC, were obtained. PCR-RFLP analysis showed one mouse (1 of 9; 11%) having cells with CYP3A5*1 or CYP3A5*3, implying mosaicism (Fig. 4B and Supplementary Fig. 3). Other mice had the CYP3A5*3 genotype (Fig. 4B and Supplementary Fig. 3). The mouse with potential CYP3A5 mosaicism was mated with a mouse-Cyp3a-KO mouse, from which five GFP-positive offspring were obtained. Fortunately, PCR-RFLP analysis revealed that three of these five mice had the CYP3A5*1 genotype and the other mice had the CYP3A5*3 genotype (Fig. 4C and Supplementary Fig. 5). These results suggest that the mouse obtained by injection did indeed exhibit mosaicism. To avoid random integration, direct sequencing was performed with primers that amplify a wider region of the homologous sequence for SNP modification. Direct sequencing using the genome of the tail of F_1_ mice as a template revealed the precise SNP modification without any other sequence rearrangement around the CRISPR/Cas9 target site (data not shown). We also obtained a positive result in further genomic PCR for other *CYP3A*s (Fig. 4D and Supplementary Fig. 4). The mice carrying the CYP3A-MAC with CYP3A5*1 were further mated with mouse-Cyp3a-KO mice, leading to the eventual production of a humanized CYP3A5*1 mouse. The humanized CYP3A5*1 mice generated via pronuclear injection were used in following studies.

### CYP3A5 protein expression in the liver and intestine of CYP3A5*1 mice

**Figure 5:**
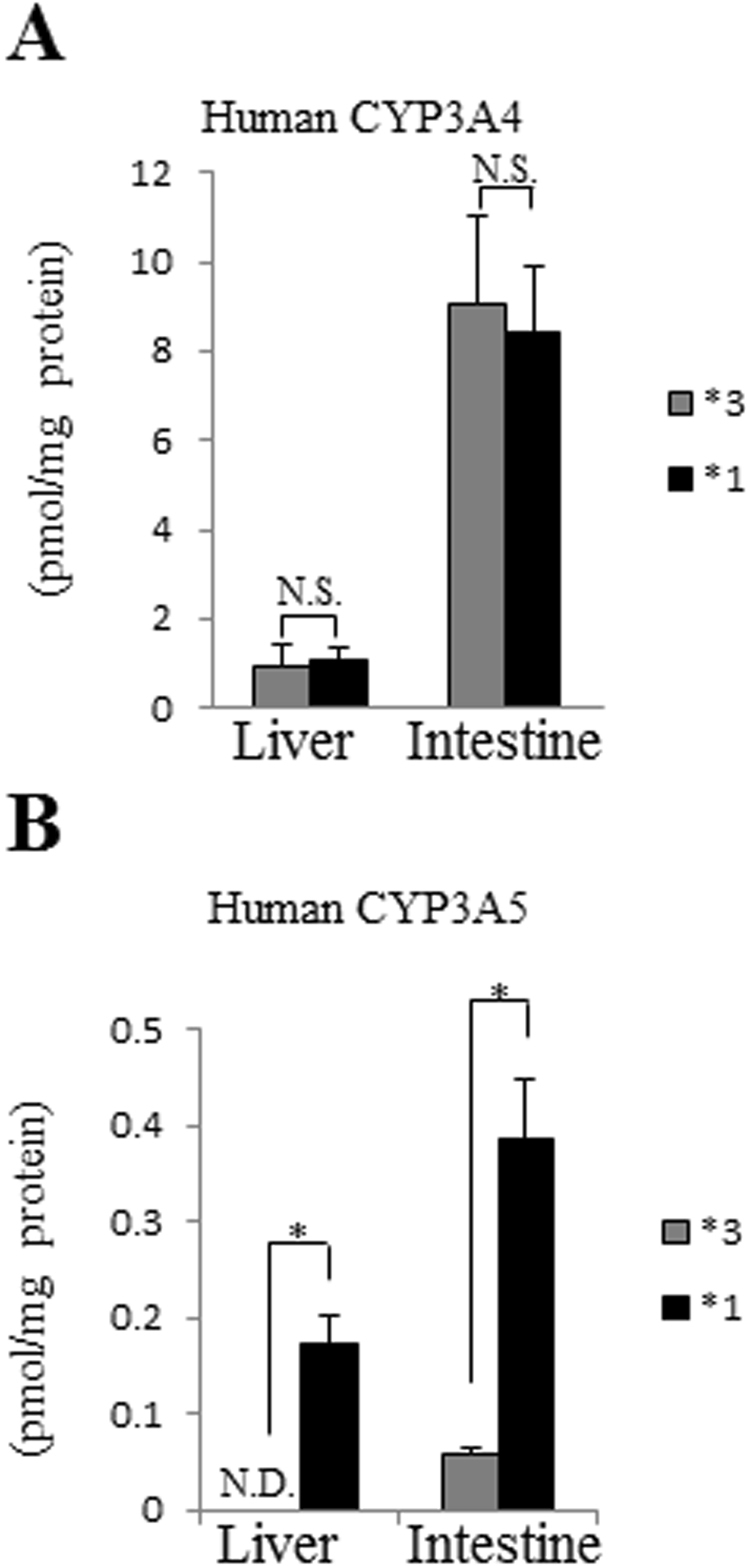
Protein quantitative analysis of CYP3A5*1 mice. (**A**) LC-MS/MS analysis results for CYP3A4 protein quantity in the liver and intestine of humanized CYP3A5*3(*3) and CYP3A5*1(*1) mice. (**B**) LC-MS/MS analysis results for CYP3A5 protein quantity in the liver and intestine of humanized CYP3A5*3(*3) and CYP3A5*1(*1) mice. Data are expressed as the means ± S.D. (n = 4 mice). N.D., not detected. N.S., not significant. *P < 0.005.

To determine the protein expression of human CYP3A derived from the CYP3A5*1 mice, an LC-MS/MS-based assay was performed. CYP3A4 expression in the liver and intestine was comparable between CYP3A5*3 and CYP3A5*1 mice (Fig. 5A). However, the amount of CYP3A5 protein in the liver and intestine was significantly higher in CYP3A5*1 mice than in CYP3A5*3 mice (Fig. 5B). These findings suggested that *CYP3A5* SNP modification increased the expression of the CYP3A5 protein.

### Metabolic activity of CYP3A in the liver and intestine of CYP3A5*1 mice

**Figure 6:**
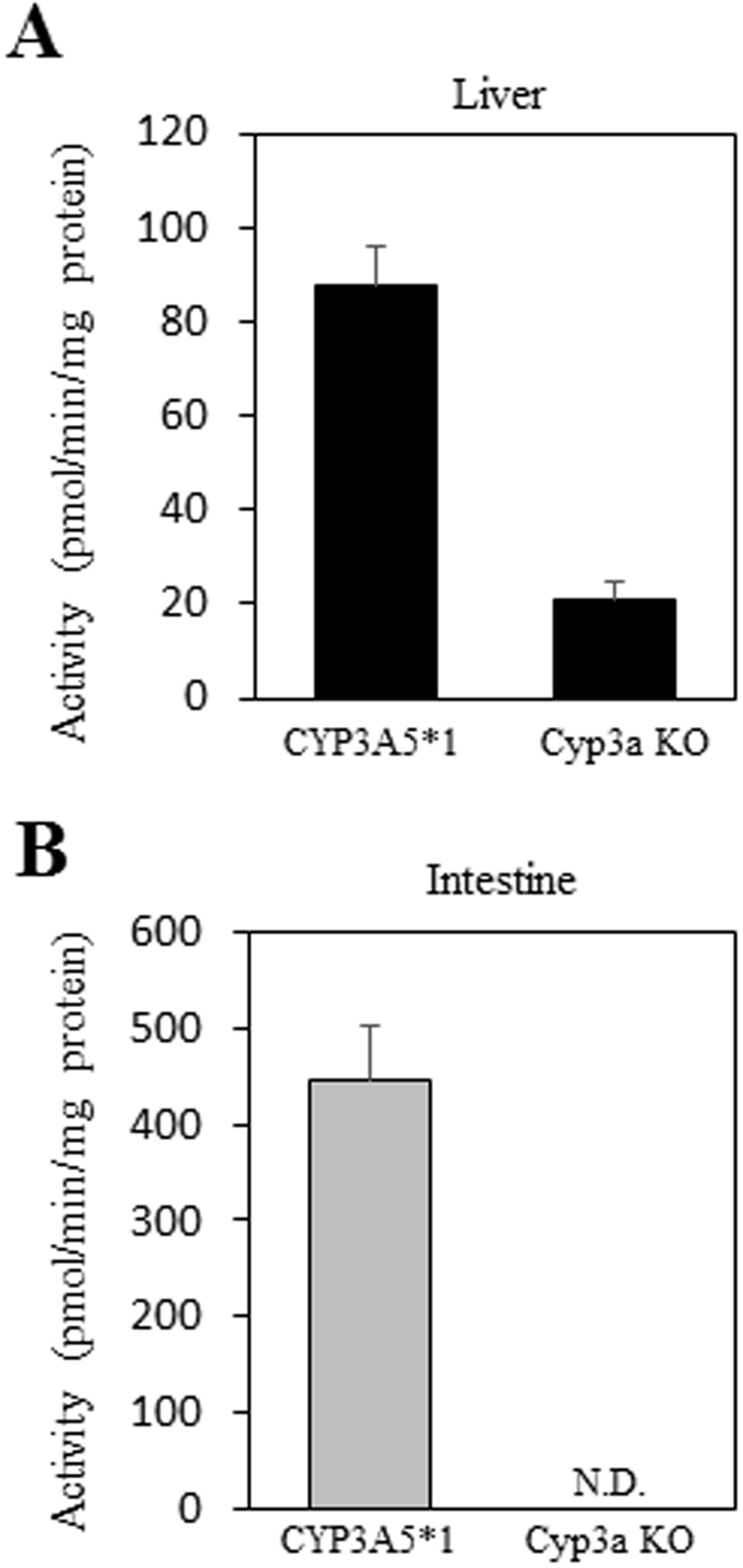
Assay of triazolam hydroxylation activity in CYP3A5*1 mice. (**A**) Triazolam alpha-hydroxylation activities in liver microsomes of Cyp3a-KO and CYP3A5*1 mice. (**B**) Triazolam alpha-hydroxylation activities in intestinal microsomes of Cyp3a-KO and CYP3A5*1 mice. Data are expressed as the means ± S.E. (n = 3 mice) of three independent assays, each performed in duplicate. N.D., not detected.

Triazolam is a widely used substrate for assessing the contribution of CYP3A4 and CYP3A5 activity to metabolism^23–26^. To investigate the function of the CYP3A gene in CYP3A5*1 mice, triazolam alpha-hydroxylation activities were analyzed in the liver and intestine of CYP3A5*1 mice. In both liver and intestinal microsomes of CYP3A5*1 mice, apparent activities were detected, which contrasted to the very low levels or absence of activity in mouse-Cyp3a-KO mice (Fig. 6A and B). These findings suggested that CYP3A genes in CYP3A5*1 mice are functional and not disrupted by off-target effects via genome editing.

### Inhibition assay for the validation of CYP3A5 contribution

**Figure 7:**
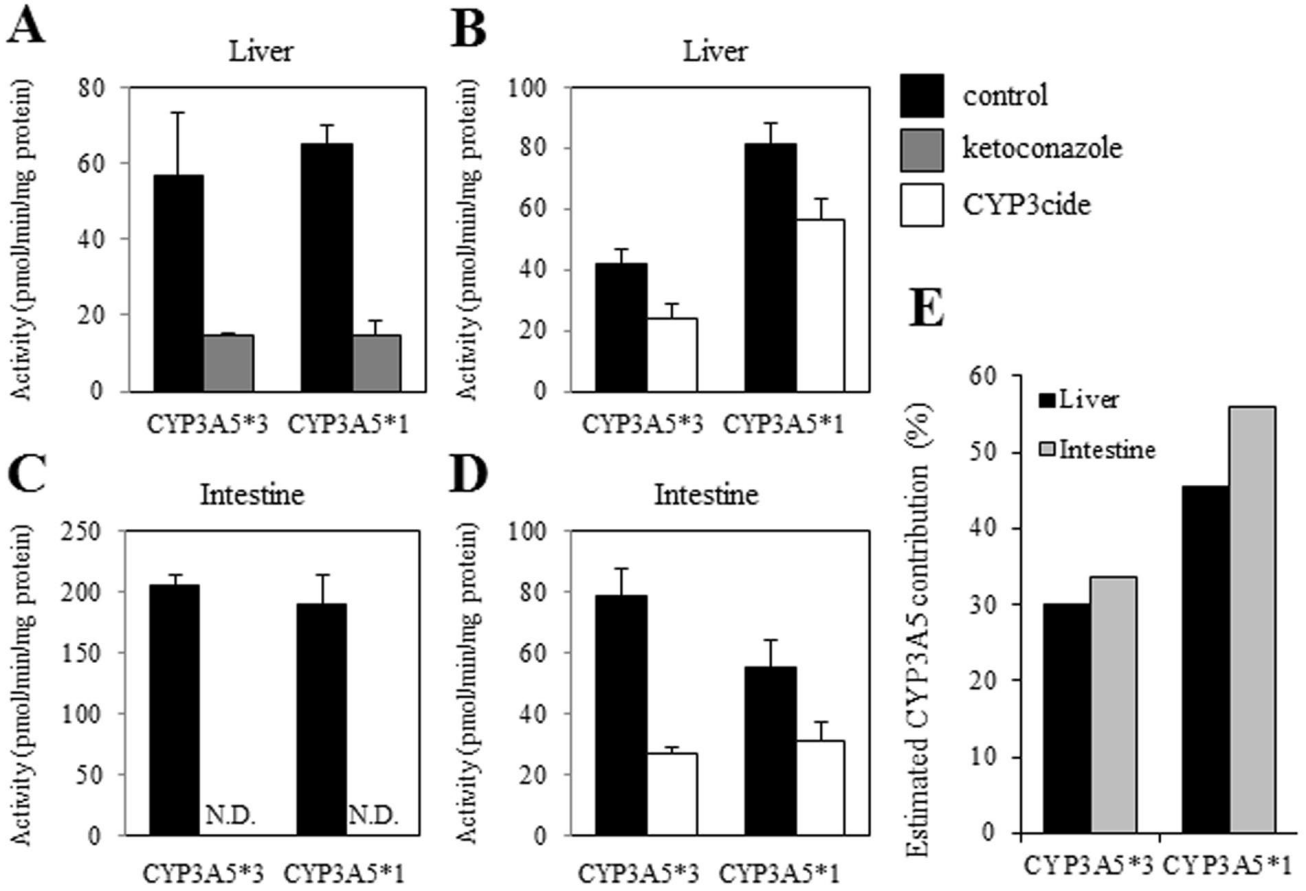
CYP3A inhibition assay for evaluating the CYP3A5 contribution in CYP3A5*1 mice. (**A**) The effect of ketoconazole on the triazolam alpha-hydroxylation activity in liver microsomes of CYP3A5*3 and CYP3A5*1 mice. (**B**) The effect of CYP3cide on the triazolam alpha-hydroxylation activity in liver microsomes of CYP3A5*3 and CYP3A5*1 mice. (**C**) The effects of ketoconazole on the triazolam alpha-hydroxylation activities in intestinal microsomes of CYP3A5*3 and CYP3A5*1 mice. (**D**) The effects of CYP3cide on the triazolam alpha-hydroxylation activities in intestinal microsomes of CYP3A5*3 and CYP3A5*1 mice. Pooled microsomes from four mice in each group were used. Data are expressed as the means ± S.D. of three independent assays, each performed in duplicate. (**E**) The estimation of CYP3A5 contribution in CYP3A5*3 and CYP3A5*1 mice. N.D., not detected.

CYP3A5 activity should be confirmed to check whether the functional CYP3A5 protein is expressed in CYP3A5*1 mice. However, it has been reported that there is no apparent difference in substrate specificity between CYP3A4 and CYP3A5^27^. In addition, no potent and selective CYP3A5 inhibitors have been identified. Thus, CYP3A4/3A5 and CYP3A4 inhibition assays were conducted to estimate the functional contribution of the CYP3A5 protein to triazolam alpha-hydroxylation, in accordance with a previously reported method^23^. Ketoconazole was used as an inhibitor of both CYP3A4 and CYP3A5, and CYP3cide was used as a selective inhibitor of CYP3A4. As shown in Fig. 7A and B, ketoconazole and CYP3cide inhibited the activities in the liver microsomes of CYP3A5*1 (76.8% and 31.2% inhibition, respectively) and CYP3A5*3 mice (71.9% and 41.9% inhibition, respectively). In the intestinal microsomes, the activities of both CYP3A5*1 and CYP3A5*3 mice were completely inhibited by ketoconazole (Fig. 7C), but the percent inhibitions by CYP3cide of the activities of CYP3A5*1 mice were smaller than those of CYP3A5*3 mice (43.9% and 66.5% inhibition, respectively, Fig. 7D). When the percent contributions of CYP3A5 protein to triazolam alpha-hydroxylation activities were estimated by subtracting the percent inhibition by CYP3cide (% CYP3A4 contribution) from the percent inhibition by ketoconazole (% CYP3A contribution), a significant CYP3A5 contribution was identified in both liver and intestinal microsomes derived from CYP3A5*1 mice (Fig. 7E). These results suggested that functional CYP3A5 protein and higher CYP3A5 activity are exhibited in CYP3A5*1 mice compared with CYP3A5*3 mice. These findings together show that CYP3A5*1 mice, developed by genome editing technology, can act as a model recapitulating a human CYP3A5*1 carrier.

## Discussion

In the present study, we successfully performed SNP modification using genome editing technology in both mouse ES cells and fertilized eggs, and produced mice with different SNPs. Humanized mouse models for analyzing the effect of SNPs have been reported, such as for *TP53*, the effect of an SNP of which was revealed in bacterial artificial chromosome (BAC) transgenic mice^28^. However, the BAC transgenic system has several potential problems in copy number control, integration site effect, and limitation of genomic size to transfer to cells. The MAC vector system solves these problems, with advantages such as desired copy delivery, independent maintenance without integration to the host genome, and no genome size limitation to deliver. The MAC vector system allows us to transfer one copy (one allele) of a gene or genome into animals; it should enable the effects of polymorphisms of the transferred human genomes to be clearly observed.

Because the coordinated expressions of CYP3A4 and CYP3A5 affect metabolic function, the expression of both in mice is necessary for the prediction of drug metabolism in humans with CYP3A5*1 allele. Thus, the modification of the CYP3A-MAC carrying both *CYP3A4* and *CYP3A5* genomic regions was best suited for this study. The comparison of CYP3A5*1 and CYP3A5*3 mice is expected to allow us to validate the contribution of CYP3A5 in CYP3A4-expressing situations for drug screening. Our model mice are the first humanized Tc mice for the prediction of the effects of SNPs on pharmacokinetics.

The CRISPR/Cas9 system allows us to modify SNPs with high efficiency in both mouse ES cells and fertilized eggs (18.8% and 11% success rates, respectively). The use of mRNA and modified Cas9, eSpCas9, or SpCas9-HF1 may increase the efficiency of such modifications by improving specificity and preventing the re-cutting of modified sequences^29,30^. In the present study, we utilized the pCAG-EGxxFP system and carefully selected a target sequence for SNP modification. Because evaluating whether the modified sequence is still targeted by CRISPR/Cas9 is very important, this system is very useful when selecting an appropriate target sequence for SNP modification.

CYP3A5 expression in the liver and intestine was higher in CYP3A5*1 mice than in CYP3A5*3 mice. Other CYP3A genes, particularly CYP3A4, were also expressed, and the metabolic activity was not disrupted, showing that designed gRNA (CYP3A5_B) was specific to CYP3A5, with no off-target effect. The CYP3A inhibition assay detected the contribution of CYP3A5 to CYP3A-mediated metabolism. These findings suggested that CYP3A5 SNP was functionally modified without the disruption of any other CYP3A gene.

The CYP3A5*3 allele causes splicing defect, and participation of the nonsense-mediated mRNA decay has been reported to be associated with the degradation of splice variants^31^. However, some homozygotes of CYP3A5*3 allele have been reported to express the CYP3A5 protein at a low level. It is unclear how some RNA is spliced normally from the CYP3A5*3 allele. The effect of *CYP3A5* SNP is actually recapitulated in mice. Therefore, it is expected to be possible to analyze the mechanism of CYP3A5 expression associated with SNPs using our models.

Various SNPs in *CYP3A4* have been reported, but their effects have mainly been evaluated *in vitro* ^32^. This is also relevant for *MDR1*, one of the most prominent and extensively studied transporters, for which polymorphisms have also been identified^33^. Despite efforts to understand the effects of *MDR1* polymorphisms on pharmacokinetics, no consensus has been reached on this issue because of the lack of a humanized animal model. Regarding the *CYP2C* family, the effects of *CYP2C9* and *CYP2C19* polymorphisms on metabolic activity toward drugs that are known substrates of these proteins have been reported^34,35^. Polymorphisms in UGT2 genes have also been reported to affect expression and metabolic activity^36^. The combined technologies, HAC/MAC and genome editing, developed in this study could allow us to create humanized models that reflect conditions in humans with different SNPs or polymorphisms with ease and speed.

A generation of Tc mice with various SNPs, starting from the development of an original human chromosome library harboring desired SNPs, is extremely hard and takes an excessive amount of time. The effective way for generation of humanized mice with desired SNPs is to modify SNPs of already constructed basic HACs/MACs carrying the desired human genome.

The present study effectively predicts the effect of polymorphisms on pharmacokinetics. Similar to the production of humanized CYP3A mice, we can produce a variety of humanized model mice, such as those with SNP reported genes, as mentioned above, and then we can modify SNPs or polymorphisms in these humanized models to produce a useful repertoire of models. In addition, the development of humanized multiple gene models with defined polymorphisms can also play an important role in understanding complex pharmacokinetics.

## Materials and Methods

### PCR-RFLP analysis of CYP3A5 SNP

PCR-RFLP analysis was performed as described previously^16^. Briefly, PCR was performed using the following primers: CYP3A5 6956Fm, 5′-CTT TAA AGA GCT CTT TTG TCT CTC A-3′, and CYP3A5 7155R, 5′-CCA GGA AGC CAG ACT TTG AT-3′. PCR products were treated with DdeI for 2 h at 37 °C before electrophoresis using 3% agarose gel.

### Plasmid construction

Sense and antisense oligonucleotides for each gRNA were annealed and inserted into a BbsI site of the pX330 plasmid expressing Cas9/gRNA. pX330-U6-Chimeric_BB-CBh-hSpCas9 was a gift from Feng Zhang (Addgene plasmid # 42230)^19^. Cloning of annealed oligonucleotides was confirmed by PCR using the following primers: human U6 promoter FW, 5′-GAG GGC CTA TTT CCC ATG ATT CC-3′, and each corresponding antisense oligonucleotide. The oligonucleotides used were as follows: CYP3A5_A S, 5′-CAC CTT TCA GTA TCT CTT CCC TGT TTG G-3′; CYP3A5_A AS, 5′-AAA CCC AAA CAG GGA AGA GAT ACT GAA A-3′; CYP3A5_B S, 5′-CAC CAA GGG TAA TGT GGT CCA AAC AGG G-3′; and CYP3A5_B AS, 5′-AAA CCC CTG TTT GGA CCA CAT TAC CCT T-3′.

To construct the pCAG-EGxxFP validation plasmid, each target and potential off-target sequence was amplified by PCR using humanized CYP3A mouse tail DNA or synthesized DNA (Life Technologies, Carlsbad, CA, USA) as a template. The sequences of each primer set were as follows: C3A5 bam_L, 5′-GCT TGG ATC CAC CAA CTG CCC TTG CAG CAT-3′, and C3A5 ecoi_R, 5′-ACG CCG AAT TCT GGG GAC AAC GGA GCT GAT T-3′, for CYP3A5*1 or *3; C3A4 bam_L, 5′-GCT TGG ATC CAT GGA GAA TGG CAT GGG AAA-3′, and C3A4 ecoi_R, 5′-ACG CCG AAT TCC CAG CAC AGG CTG TTG ACC A-3′, for CYP3A4; C3A7 bam_L, 5′-GCT TGG ATC CCC CAC AGC AAC TGC CCT TGA AA-3′, and C3A7 ecoi_R, 5′-ACG CCG AAT TCG ATC TGT GAT AGC CAG CAT AGG CT-3′, for CYP3A7; and C3A43 bam_L, 5′-GCT TGG ATC CAG CCA CTG CAC CCA GCC TAT-3′, and C3A43 ecoi_R, 5′-ACG CCG AAT TCA GCC TGT GTG GGG GAA AAC A-3′, for CYP3A43. Amplified PCR products were treated with BamHI and EcoRI and ligated to BamHI- and EcoRI-digested pCAG-EGxxFP plasmid.

### Validation of cleavage activity

The cleavage activity of pX330-sgRNA was validated by co-transfection with each pCAG-EGxxFP-target plasmid into HEK293T cells, as reported previously^21^. pCAG-EGxxFP-Cetn1 and pX330-sgRNA were transfected as the positive control. Only pCAG-EGxxFP-Cetn1 was transfected as the negative control.

### SNP modification in mouse ES cells containing the CYP3A-MAC

Mouse ES cells with the CYP3A-MAC and the mice with the CYP3A-MAC were generated using the same method to construct mouse ES cells with the CYP3A-HAC and the mice with the CYP3A-HAC (Kazuki Y. *et al*., unpublished data)^2^. A total of 1 × 10^3–4^ ESCs containing the CYP3A-MAC were seeded on mouse embryonic fibroblasts in a six-well plate and transfected with pX330-A plasmids (1.0 μg) and a donor plasmid (1.0 μg) containing a synthesized sequence for SNP modification (Life Technologies, Carlsbad, CA, USA), using Lipofectamine LTX & PLUS reagents (Life Technologies, Carlsbad, CA, USA)^22^. The picked clones were analyzed using PCR-RFLP.

### Genomic PCR analysis

Primer pairs for the detection of a region of the human CYP3A cluster and genotyping of mouse-Cyp3a-KO were as described previously ^2^. Primer sets for the direct sequencing of the modified region of CYP3A5 were as follows: C3A5 int3 L, 5′-GGT GCC CTT TTA TCA CAT GCA TTG TCT C-3′, and C3A5 int4 R, 5′-TCA TTG GGT TGC CCA GAC TGG AGT ATA A-3′.

### Ethics statement

All experimental procedures were approved by the Institutional Animal Care and Use Committee of Tottori University. Methods were conducted in accordance with the approved guidelines of the Institutional Animal Care and Use Committee of Tottori University.

### Pronuclear injection

ICR female mice were superovulated and mated with CYP3A-MAC males, and fertilized eggs were collected. A circular pX330-A plasmid was injected into one of the pronuclei at 5 ng/μL with ssODN at 100 ng/μL. The sequence of ssODN is 5′-AAC GAA TGC TCT ACT GTC ATT TCT AAC CAT AAT CTC TTT AAA GAG CTC TTT TGT CTT TCA ATA TCT CTT CCC TGT TTG GAC CAC ATT ACC CTT CAT CAT ATG AAG CCT TGG GTG GCT CCT GTG TGA GAC TCT TGC-3′.

### Chimeric mouse production

Chimeric mice were produced using mouse ES cells with the CYP3A5*1-MAC. Briefly, ES cells were injected into eight-cell stage embryos derived from ICR mice (CLEA, Tokyo, Japan) and then transferred into pseudopregnant ICR females.

### Humanized CYP3A5*1 mouse production

A mouse with mosaicism, with cells carrying CYP3A5*1 or CYP3A5*3-MAC, was mated with a mouse-Cyp3a-KO mouse. The germline transmission of CYP3A5*1 or CYP3A5*3-MAC was monitored by EGFP expression from the MAC. Mice with the CYP3A5*1-MAC were further crossed with mouse-Cyp3a-KO mice. After further mating, humanized CYP3A5*1 mice were obtained.

### Preparation of microsomes

Liver tissue and part of the small intestine (a 10-cm segment from the upper part of the small intestine) were used for the preparation of microsomal fractions.

### Quantitative analysis of CYP3A5 protein by LC-MS/MS

The expressed CYP3A4 and CYP3A5 were digested by trypsin after reduction and cysteine blocking, after which the samples were analyzed by TOF-MS. A surrogate peptide from the tryptic digestion of CYP3A4 and CYP3A5 was confirmed by TOF-MS, and its characteristic spectrum was collated using the protein sequence database UniProtKB/Swiss-Prot. Its identified spectrum as each CYP3A4 and CYP3A5 was used for constructing a measurement method by LC-MS/MS. Calibration curves prepared from the samples were spiked with expressed CYP3A4 and CYP3A5. Liver and intestinal microsomes (10 μL, 10 mg/mL) were digested in a similar manner and were quantified.

### Assay of triazolam hydroxylation activities

The basic incubation mixture contained liver or intestinal microsomes, 100 mM potassium phosphate buffer (pH 7.4), 0.1 mM EDTA, NADPH generating system, and 200 µM triazolam (Wako Pure Chemicals, Osaka, Japan) as a CYP3A substrate. Incubation was conducted at 37 °C for 30 min (intestine) or 60 min (liver) and terminated by adding acetonitrile. After centrifugation, the supernatant was analyzed by HPLC.

### CYP3A inhibition assay

A CYP3A4/3A5 inhibitor, ketoconazole (2 µM; Sigma-Aldrich, St. Louis, MO, US), was co-incubated with triazolam in the reaction mixture. For CYP3A4 inhibition, microsomes were incubated with triazolam for 30 min after pre-incubation with CYP3cide (2.5 µM; Sigma-Aldrich, St. Louis, MO, US), an inhibitor of CYP3A4, for 20 min in the reaction mixture.

## Electronic supplementary material

Supplementary information Kazuki
