# Supplementary material for: Modification of single-nucleotide polymorphism in a fully humanized CYP3A mouse by genome editing technology
Source: Sci Rep. 2017 Nov 9;7:15189. doi: 10.1038/s41598-017-15033-0 (PMC5680201; doi:10.1038/s41598-017-15033-0)
Supplement: Supplementary file 1 — Supplementary information Kazuki [file 41598_2017_15033_MOESM1_ESM.pdf]

Supplementary information

Modification of single-nucleotide polymorphism in a fully humanized CYP3A mouse by genome editing technology

Satoshi Abe[1], Kaoru Kobayashi[2], Asami Oji[3], Tetsushi Sakuma[4], Kanako Kazuki[1], Shoko Takehara[1], Kazuomi Nakamura[5], Azusa Okada[2], Yasuko Tsukazaki[6], Naoto Senda[1,6], Kazuhisa Honma[7], Takashi Yamamoto[4], Masahito Ikawa[3], Kan Chiba[2], Mitsuo Oshimura[1], Yasuhiro Kazuki[1,7*]

[1]Chromosome Engineering Research Center (CERC), Tottori University, 86 Nishi-cho, Yonago, Tottori 683-8503, Japan
[2]Graduate School of Pharmaceutical Sciences, Chiba University, 1-8-1 Inohana, Chuo-ku, Chiba 260-8675, Japan
[3]Research Institute for Microbial Diseases, Osaka University, Suita, Osaka 565-0871, Japan
[4]Department of Mathematical and Life Sciences, Graduate School of Science, Hiroshima University, Higashi-Hiroshima 739-8526, Japan
[5]Division of Laboratory Animal Science, Research Center for Bioscience and Technology, Tottori University, 86 Nishi-cho, Yonago, Tottori 683-8503, Japan
[6]Tsukuba Bioanalytical Laboratory, Shin Nippon Biomedical Laboratories, Ltd., 2-1-6 Sengen, Tsukuba, Ibaraki 305-0047, Japan
[7]Department of Biomedical Science, Institute of Regenerative Medicine and Biofunction, Graduate School of Medical Sciences, Tottori University, 86 Nishi-cho, Yonago, Tottori 683-8503, Japan

[*]Correspondence should be addressed Y.K. (kazuki@grape.med.tottori-u.ac.jp).
Chromosome Engineering Research Center, Tottori University, 86 Nishi-cho, Yonago, Tottori 683-8503, Japan, Phone: +81-859-38-6219, Fax: +81-859-38-6210

Supplementary Figure 1

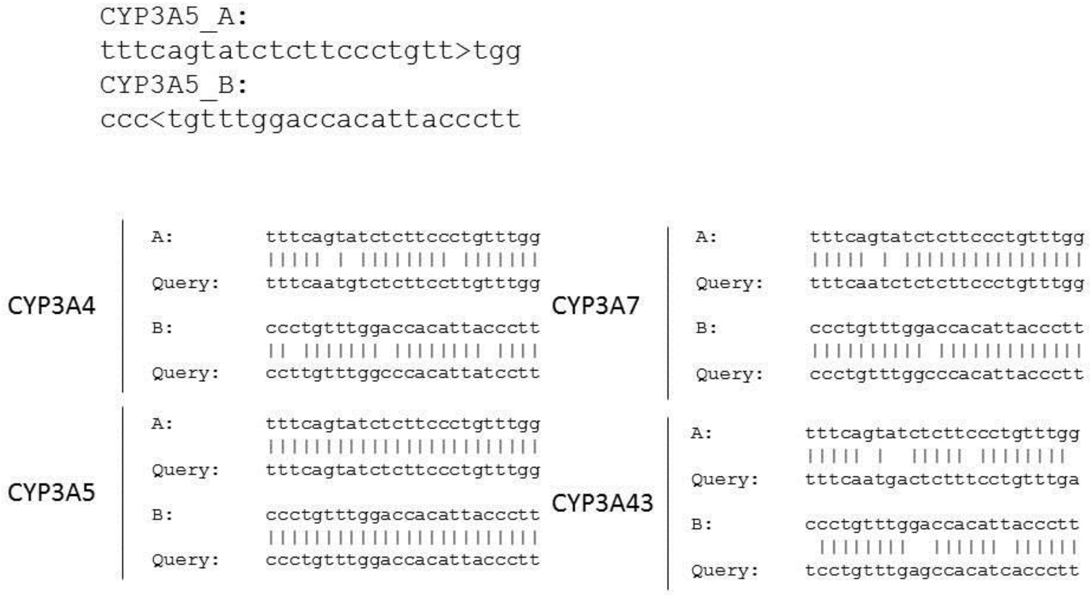

Supplementary Figure 1. Potential off-targets by CRISPR/Cas9 system.

Each potential off-target sequence in CYP3A genes for CYP3A5_A and CYP3A5_B is shown.

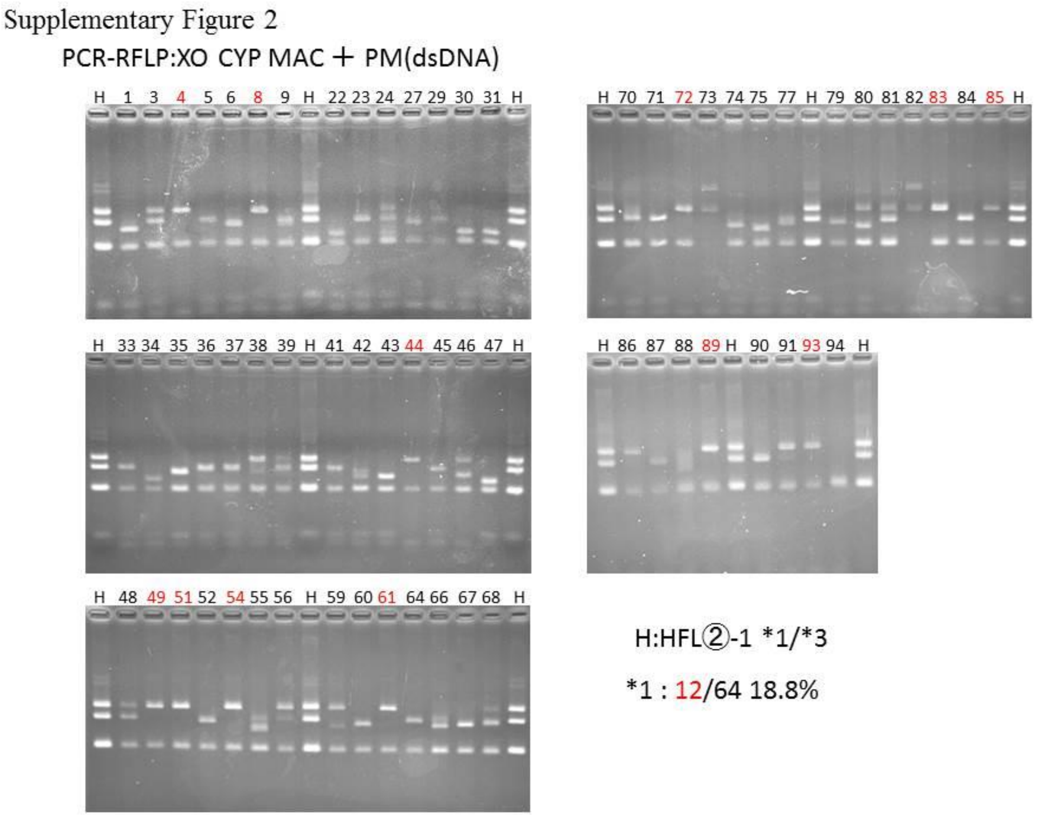

Supplementary Figure 2. PCR-RFLP analyses of mouse ES cells carrying the CYP3A-MAC which were obtained by ESC-transfection.

PCR-RFLP results for the screening of ES cells carrying the CYP3A5*1-MAC.

H: control carrying both CYP3A5*1 and CYP3A5*3 alleles.

Supplementary Figure 3

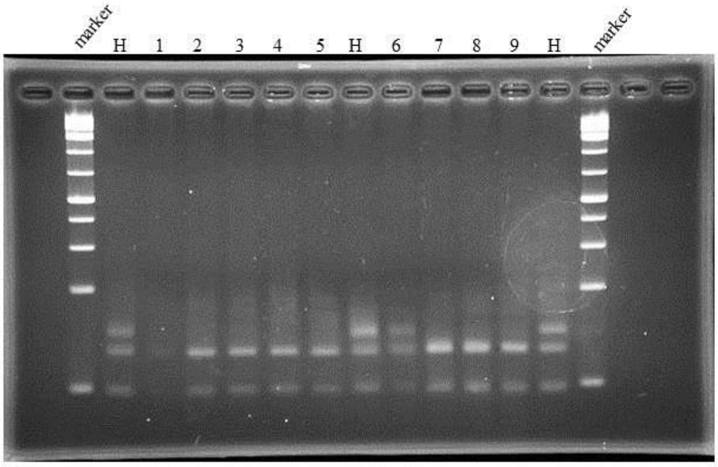

Supplementary Figure 3. PCR-RFLP analysis of F_0 mice for CYP3A5 SNP.

PCR-RFLP analysis result for the screening of F_0 mouse carrying the CYP3A5*1-MAC.

H: control carrying both CYP3A5*1 and CYP3A5*3 alleles.

Supplementary Figure 4

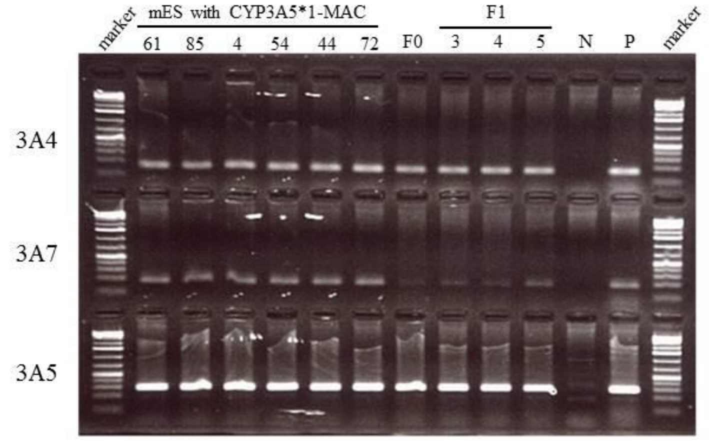

Supplementary Figure 4. PCR analysis for each CYP3A gene on the CYP3A5*1-MAC.

Mouse ES cells carrying CYP3A5*1-MAC obtained by ESC-transfection and F_0 and F_1 mouse derived via pronuclear injection was analyzed by PCR for each CYP3A gene. N: ICR mouse tail DNA, P: Tail DNA of mouse carrying the CYP3A5*3-MAC.

Supplementary Figure 5

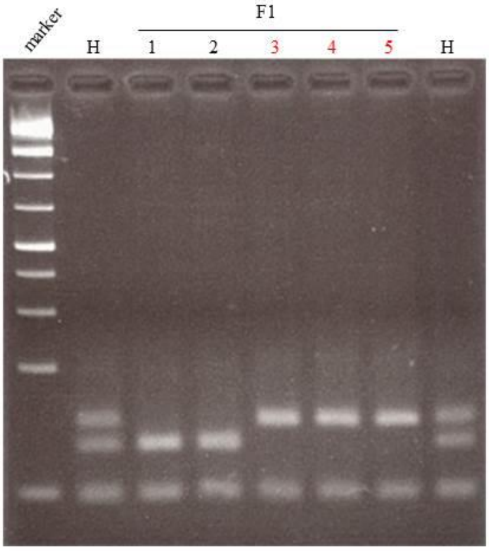

Supplementary Figure 5. PCR-RFLP analysis of F_1 mice carrying the CYP3A5*1 or CYP3A5*3-MAC derived from F_0 mosaic mouse obtained via pronuclear injection.

PCR-RFLP analysis was performed using the tail genome DNA of F_1 mice as template. H: control carrying both CYP3A5*1 and CYP3A5*3 alleles.
